# Assessment of paper dust exposure and chronic respiratory symptoms among paper factory workers in, Ethiopia; a comparative cross-sectional study

**DOI:** 10.1186/s12890-023-02338-2

**Published:** 2023-02-01

**Authors:** Bereket Meskele Negash, Samson Wakuma Abaya, Teferi Abegaz, Abera Kumie Takele, Worku Tefera Mekonnen, Hager Badima Negatu, Tamene Tesema Gintamo, Teshome Tamirat, Gelaneh Kusse Koirita

**Affiliations:** 1 Addis Ababa City Administration Health Bureau, Addis Ababa, Ethiopia; 2 Department of Preventive Medicine, School of Public Health, College of Health Sciences, Addis Ababa University, Addis Ababa, Ethiopia; 3 St. Paul’s Hospital Millennium Medical College, Addis Ababa, Ethiopia

**Keywords:** Paper dust exposure, Chronic respiratory symptoms, Paper worker, Ethiopia

## Abstract

**Background:**

Workers in pulp and paper factories are continuously exposed to paper dust. Excessive exposure to paper dust can cause respiratory disease. Information about the prevalence of chronic respiratory symptoms and dust exposure levels among workers in pulp and paper factories is not available in Ethiopia. The aim of this study was, therefore, to assess personal total dust exposure levels, the prevalence of chronic respiratory symptoms and their associated risk factors among workers in Ethiopian pulp and paper factories.

**Methods:**

A comparative cross-sectional study was conducted. A total of 40 dust measurements were carried out on 20 randomly selected workers. To assess chronic respiratory symptoms and associated factors, 434 workers from two paper factories and controls were interviewed using a standard questionnaire adapted from the American Thoracic Society (ATS). Gravimetric analyses of the filters were undertaken using a standard microbalance. Poisson regression was performed for comparing the prevalence of symptoms and risk factors for the two groups. Multivariable analyses were conducted to identify factors associated with chronic respiratory symptoms.

**Result:**

The arithmetic mean (AM) and geometric mean (GM) of dust exposure levels among the paper factories workers were 11.3 (± 7.7) and 10.2 (± 1.4) mg/m^3^ respectively. This exposure level exceeded the threshold limit value recommended for total dust (10 mg/m^3^). The prevalence of having at least one chronic respiratory symptom was about 51% among the workers in paper factories. The prevalence ratio of having chronic respiratory symptoms among paper factory workers was 5.6 times higher (PR = 6, 95% CI 3.5–10.3) than in the controls. Chronic respiratory symptoms were significantly associated with factors such as an educational status of less than grade 9, being employed in the work sections of the factories, having work experience of 5 years and above, working more than 8 h per day and having a past history of occupation and respiratory illnesses.

**Conclusion:**

The dust concentration in the paper factories exceeded the acceptable recommended limit value of 10 mg/m3. The prevalence of chronic respiratory symptoms among paper factory workers was higher than among controls. The main determining factors for chronic respiratory symptoms among the workers were the specific work section such as production section, low income, having past history of respiratory illnesses, the number of years of working and low educational status. This finding indicated the need for improving the working conditions in paper factories in Ethiopia.

**Supplementary Information:**

The online version contains supplementary material available at 10.1186/s12890-023-02338-2.

## Background

Papermaking mainly uses tree trunks, which pass through the processes of cutting, debarking, chipping and pulping. Currently, recycled paper accounts for an increasing proportion of paper production. Dust is generated at each stage of the paper-making process [1–6].

Workers in paper factories are often exposed to paper dust. Many studies have shown that the level of dust exposure in paper factories has been above recommended occupational exposure limits (OEL) of 10 mg/m^3^ [7–10]. However, some studies conducted in paper factories also indicated low levels of dust exposure [11–14].

Exposure to paper dust in excess of 10 mg/m^3^ over a period of many years can cause obstructive and restrictive respiratory diseases such as chronic obstructive lung disease, asthma and reduced lung function (mainly decreased forced expiratory volume by one second (FEV_1_) and forced vital capacity (FVC)) [7, 9, 12, 14–16]. Another study indicated that excessive exposure to paper dust (> 5 mg/m^3^) was associated with decreased lung function and increased prevalence of different forms of respiratory symptoms [12]. The development of occupational asthma or asthma-like symptoms is probably a significant occupational health hazard in pulp mills [7, 12]. Generally, the health effects vary depending on the types of dust, the duration of the exposure, and the concentration and size of dust in the breathing zone [17].

The effect is higher in those factories that use waste paper as their raw material because this production process generates a huge amount of dust [7]. In addition, dust exposure levels are higher in factories that have old machines. This can increase the impact on respiratory health [15]. By comparison, there was no correlation between exposure to paper dust and impairment of lung function in two studies of lower exposure levels (≤ 5 mg/m^3^) [18, 19]. This suggests that respiratory problem is associated with the amount of dust exposure.

Occupational respiratory symptoms and diseases are a major burden in low and middle-income countries [20]. This is a result of poor working practice and environments, and the use of outdated machines that can create excessive dust [20]. Workers in the paper industry will be likely to increase in Africa particularly in Ethiopia due to economic growth [21].

Several studies have been performed in Sweden [11–16, 19, 22–24], Germany [8, 9], and Poland [25] concerning paper dust. However, to the best of our knowledge there is no published scientific study on the level of worker exposure to paper dust in Africa particularly in Ethiopia. In addition, the paper factories in Ethiopia have long service years, more than 60 years, and were established in the 1960s. So, it seemed likely to involve high levels of dust exposure that might lead to different respiratory health problems among the paper factories workers here. Therefore, the aim of this study was to assess personal total dust exposure level, the prevalence of chronic respiratory symptoms, and their associated factors among workers in paper factories in Ethiopia.

## Methods

### Study area, period and design 

The study was conducted in two paper factories in Ethiopia (factories A, and B). The study focused on paper factories that use the material pulp, and recycled old paper as their input and which produced rolled and finished paper [26, 27]. A comparative cross-sectional study was conducted from September 2020 to October 2020 to assess personal dust exposure level, the prevalence of chronic respiratory symptoms, and their associated factors among workers of paper and water bottling factories in Ethiopia. In this study, water bottling factories were used as a control, assuming that there is less (insignificant) amount of dust generation in such factories [28].

### Source and study population

All paper factory workers and all water-bottling factories found in Ethiopia were the source population. The study populations included workers selected from these factories.

### Selection criteria

Workers who were directly engaged in the production activities in the factories for at least one year or more were included in this study. Those workers less than 18 years of age were excluded from the study.

### Sample size determination

The sample size for personal dust exposure assessment was calculated based on the Rappaport et al. 2008 recommendation that 5–10 randomly selected individuals in a similar exposure group (SEG) with repeated measurements are sufficient to predict the group dust exposure level [29]. A SEG is a group of workers working on the same type of activities in a similar work area for the same duration of time. With the above assumptions, 4 SEGs were identified from two selected factories. In factory A there were raw material preparation workers (bell machine compactor and loader, and pulp and recycle paper cutting and milling) as well as production room workers (roll paper, carton and paper production) and in factory B, there were Cone production workers and production room workers (roll paper, carton and paper). Five workers were randomly selected from each SEG. This makes a total of 40 dust measurements on 20 randomly chosen paper factory workers with each a repeated measurement.

The sample size for respiratory symptoms was calculated using a double population proportion formula considering the prevalence of coughs among exposed 36.6% and controls 18.4% [10]. An 80% statistical power was considered sufficient to detect the difference in coughing between the two groups at a significance level of 0.05. To maximize the sample size with an odds ratio of 2.56, we recalculated by taking an odds ratio of 2 on Epi info. After adding 10% for non-response, we determined that 434 participants were needed (i.e., 217 from paper factories and 217 from water bottling).

### Sampling technique

The calculated sample size was proportionally assigned to the size of the two selected factories. By using worker registrations as a sampling frame, a simple random sampling method was used to select study subjects from each department.

### Data collection procedure

#### Total dust measurements 

Personal total dust was sampled using pre-weighed polyvinyl chloride (PVC) filter membranes with a pore size of 5.0 μm inserted on a Millipore plastic closed face 37 mm filter cassette (CFC) attached to an SKC sidekick pump through which air was pumped by a rechargeable battery-powered motor at a constant flow rate of 2.0 l/min. The sampling cassettes were situated on shoulder straps as close to the breathing zone as possible. Full-shift exposure measurements were conducted on randomly chosen days and repeated sampling was conducted the next day. Data collection took 4 days in each factory. While dust sampling, observation checklists and data sheets were used to record relevant data.

#### Interviews 

The chronic respiratory symptoms among participants were assessed with face-to-face interviews using a standardized structured questionnaire adapted from the American Thoracic Society (ATS) [30]. The standardized questionnaire includes characteristics of participants’ factors, duration of exposure, previous occupational history, smoking habits, as well as respiratory symptoms such as cough, phlegm, wheezing, shortness of breath, and nose irritations, particularly those associated with the risk of respiratory morbidity.

#### Observational checklist 

An observational checklist was used to assess the working condition (including housing conditions, ventilation, type of machine, personal protective equipment (PPE) used and general working conditions).

### Data analysis

The result of the dust concentration was described using descriptive statistics such as the measure of central tendency (GM) and the measure of dispersion (GSD). The exposed filter membrane was weighed quantitatively by gravimetric analysis using a standard Mettler Toledo XPE105 micro balance scale with a detection limit of 0.01 mg at the Environmental and Occupational Health laboratory of the College of Health Sciences, Addis Ababa University. The net concentration was calculated by weighing the sampling filter before and after sampling.

$${\text{Concentration }}\left( {{\text{mg}}/{\text{m}}^{{3}} } \right) \, = \frac{{\left( {{\text{post weight of filter }}{-}{\text{pre}} - {\text{weight of filter}}) \, \left( {{\text{mg}}} \right) - {\text{blank weight }}\left( {{\text{mg}}} \right)} \right)}}{{{\text{Flow rate }}\left( {\text{lit per min}} \right) \, \times {\text{ sample duration }}\left( {{\text{min}}} \right) \, \times {1}/{1}000{\text{ m}}^{{3}} /{\text{lit}}}}$$

The average dust concentration was compared with the threshold limit values (TLV) for total dust (also denoted as nuisance dust or Particles Not Otherwise Specified (PNOS), recommended by the Control of Substances Hazardous to Health Regulations 2002 (COSHH) for an 8-h exposure (10 mg/m^3^) [31]. Poisson regression models with a Robust estimator were used to estimate the prevalence ratio of different respiratory symptoms among both the workers in paper factories and controls.

Logistic regressions were used to identify factors associated with chronic respiratory symptoms. In the model chronic respiratory symptoms was used as the dependent variable and characteristics of participants, work-place, and behavioral characteristics were used as the independent variable. Only variables with *P*-value < 0.2 in the binary logistic analysis were transferred to multivariate analysis. The final model contained only variables with *P*-value ≤ 0.05. The analysis was done using the statistical software SPSS version 22.

## Data quality assurance

For dust sampling, the airflow rate in the sampling pumps was measured/checked and recorded before and after each sampling event using a Rota-meter (i.e., flow rates more than ± 10% different from the target flow rate of 2.0 lit/min were dropped). Each series of sampling was controlled and corrected towards one field blank sample daily. The sample head position was mounted based on the person’s working position. At the end of sampling, the filter cassettes were covered and carefully placed in a labeled container to prevent damage. They were then transported to the lab for analysis.

A standardized questionnaire modified from ATS was used to assure data quality. Before data collection, two days of training was given to the data collectors and supervisors in order to ensure that the questionnaires were completed appropriately and to reduce bias. In addition, the questionnaire was translated from English to Amharic and back to English to check its consistency. Each day, the supervisor checked each questionnaire for completeness and consistency. In addition, pre-tests were carried out a week before the actual data collection to check the competency of the data collectors, as well as the reliability and validity of the data collection tools.

## Variable measurements

### Outcome variables

Chronic respiratory symptoms: cough, cough with sputum, breathlessness, wheezing, or a chest illness that lasts at least three months in one year [32].

### Exposure variables

Paper dust: refers to dust coming from finely milled or otherwise processed paper and pulp products [13].

Characteristics of participants: age, sex, marital status, religion, educational status, income level and past history of respiratory illness (is a chronic respiratory diagnoses that occurred before paper dust exposure).

Family and behavioral factors: cigarette smoking, utilization of personal protective equipment (PPE), and energy source used at home for food preparation.

Work-place (environment) characteristics: ventilation, dustiness of working department, duration of employment, length of working hours/day, length of working days/week, past history of dust exposure, and type of machine.

Organizational factors: PPE provision, Occupational Health and Safety (OHS) training (formal short-term training that a worker shall have while he/she is on job) and OHS supervision (is the act of overseeing something or somebody’s activities or guide (correct) the employees if he/she is doing wrong.)

## Results

### Characteristics of participants

A total of 411 workers in paper factory and control groups participated in the study resulting in a response rate of 94.7%. Of the 411 respondents, 221 (53.8%) were male. The majority of the participants were younger than 25 with the mean (± SD) age being 28.8 (± 7.1) years. Almost half 200 (48.7%) of the study participants were married and 195 (47.5%) of the respondents had attended secondary school. The mean (± SD) monthly incomes of respondents were 3704 (± 2388) Ethiopian birr which is much below the national mean monthly income, 5090 Ethiopian birr. Fifty (12.2%) had a past history of a respiratory diseases that was confirmed by physician. In general, the distribution of sex, age, marital status, educational status, monthly incomes and past history of respiratory illnesses of the two comparative groups of paper and controls were significantly different (Table 1).

**Table 1:** Characteristics of participants of the paper and water bottling factories workers (*N* = 411)

Variables	Paper factories *N* = 206 N (%)	Controls *N* = 205 *N* (%)	Total *n* = 411 *N* (%)	*p*-value
*Sex*				
Male	130 (63.1)	91 (44.4)	221 (53.8)	
Female	76 (36.9)	114 (55.6)	190 (46.3)	< 0.001^a^
*Age (in year)*				
18–25	66 (32.0)	100 (48.8)	166 (40.4)	
26–34	67 (32.5)	93 (45.4)	160 (38.9)	
≥ 35	73 (35.)	12 (5.9)	85 (20.7)	< 0.001^a^
Mean (± SD)	31.2 (± 8.6)	26.36 (± 4)	28.8 (± 7.1)	< 0.001^b^
*Marital status*				
Single	84 (40.8)	87 (42.4)	171 (41.6)	
Married	97 (47.1)	103 (50.2)	200 (48.7)	
Widowed/Widower	25 (12.1)	15 (7.3)	40 (9.7)	0.255^a^
*Educational status*				
Primary school	35 (17)	18 (8.8)	53 (12.9)	< 0.001^a^
Secondary school	78 (37.9)	117 (57.1)	195 (47.5)	
More than 12 years of education	93 (45.2)	70 (34.2)	163 (39.7)	
*Monthly income*				
< 4000	152 (73.8)	54 (26.3)	206 (50.1)	
≥ 4000	54 (26.2)	151 (73.7)	205 (49.9)	< 0.001^a^
Mean (± SD)	3168 (± 2953)	4243 (± 1453)	3704 (± 2388)	< 0.001^b^
*Past history of respiratory illnesses*				
Yes	37 (17.96)	13 (6.34)	50 (12.2)	< 0.001^a^
No	169 (82.04)	192 (93.66)	361 (87.8)	

Number of participant (*N*); ^a^ = *χ* ^2^ test; ^b^ = independent sample *t*-test; SD Standard deviation, significant level at *p* < 0.05; controls were employed in the water bottling factories

### Work-related and behavioral characteristics 

Out of the 411 respondents, 290 (70.6%) had less than five years of work experience and the majority 224 (54.5%) of the study participants worked above eight hours per day. Three hundred ninety-nine (97.1%) of the respondents had worked more than five days per week and 43 (10.5%) of workers had exposure in previous employments in dusty areas. More than half (56.2%) of the respondents was used bio-fuel (charcoal, wood and gas) as the primary energy source for cooking at home. Only 12 (2.9%) of the participants smoked cigarettes (Table 2).

**Table 2:** Work-place characteristics and behavioural factors of the paper and water bottling factory workers (*N* = 411)

Variables		Paper factories (*N* = 206) N (%)	Controls (*N* = 205) *N* (%)	*p*-value
Work experience (in year)	< 5 5–9 ≥ 10 Mean (± SD)	102 (49.5) 45 (21.9) 59 (28.6) 7.9 (± 7.2)	188 (91.7) 17 (8.3) 0 3 (± 1.7)	< 0.001^a^ < 0.001^b^
Working hours per day	≤ 8 > 8 Mean (± SD)	187 (90.8) 19 (9.2) 8.33 (± 1.2)	0 (0) 205 (100) 10.87 (± 0.9)	< 0.001^a^ < 0.001^b^
Working days per week	≤ 5 > 5 Mean (± SD)	12 (5.8) 194 (94.2) 6.19 (± 0.5)	0 205 (100) 6.8 (± 0.4)	< 0.001^a^ < 0.001^b^
Exposure in previous employments	Yes No	28 (13.6) 178 (86.4)	15 (7.3) 190 (92.7)	< 0.05^a^
Work section Recycle paper cutting Bell machine compactor and loader Pulp and recycle paper milling Roll paper production Paper and carton production Cone production		40 (19.4) 11 (5.3) 9 (4.4) 23 (11.2) 96 (46.6) 27 (13.1)		
Bio-fuel energy use	Yes No	141 (68.5) 65 (31.6)	90 (43.9) 115 (56.1)	< 0.001^a^
Cigarette smoking behavior	Current smoking past smoker Never smoker	4 (1.9) 3 (1.5) 199 (96.6)	5 (2.4) 0 200 (97.6)	< 0.21^a^
Use/wear of PPE	Yes No	16 (7.8) 190 (92.2)		
Type of PPE used Mask respiratory Full face pieces respiratory Breathing apparatus		13 (6.3) 0 3 (1.5)		
Reasons for not using PPE Not available Not comfortable for work Not provided by institution The dust is not harmful		6 (2.9) 4 (1.9) 175 (97.6) 5 (2.4)		
OHS training	Yes No	79 (38.4) 127 (61.7)	139 (67.8) 66 (32.2)	< 0.001^a^
Supervision on OHS issues	Yes No	48 (23.3) 158 (76.7)	84 (41) 121 (59)	< 0.001^a^

Number of participant (*N*); a = *χ* ^2^ test; b = independent sample *t*-test; SD = standard deviation; significant level at *p* < 0.05; bio fuel (charcoal, wood and gas); controls were employed in the water bottling factories

Only 16 (7.8%) workers involved in this study used personal protective equipment (PPE). Whereas the remaining were not use PPE the main reasons mentioned by 175 (97.6%) of the participants were not provided by the institution and the remaining 5 (2.4%) thinks as dust is not harmful. Seventy nine (38.4%) paper factory workers and 139 (67.8%) water bottling factory workers had training on occupational health and safety (OHS) (Table 2).

### Personal dust exposure of paper factories workers 

The overall arithmetic mean (± SD) and geometric mean (± GSD) of the dust concentration were 11.3 (± 7.7) and 10.2 (± 1.4) mg/m^3^ respectively, ranging from 6.8 to 55.8 mg/m^3^. Out of 40 dust measurements 15 (37.5%) exceeded the acceptable recommended limit value of 10 mg/m^3^ of total dust of COSHH (Table 3).

**Table 3:** Personal total dust exposure levels by work section among paper factory workers (*N* = 40)

Work section (Task)	Range (mg/m^3^)	Mean (± SD) (mg/m^3^)	GM (± GSD) (mg/m^3^)	*N* above OEL (10 mg/m^3^)
*Raw material preparation*				
(Factory A)	6.8–14.5	10 (± 2.8)	9.7 (± 1.3)	3 (30%)
*Production room*				
Factory A	7.5–16	11.3 (± 2.9)	11 (± 1.3)	7 (70%)
Factory B Both Factory A and B	7.3–55.8 7.3–55.8	15.1 (± 14.7) 13.2 (± 10.5)	12 (± 1.9) 11.5 (± 1.6)	5 (50%) 12 (60%)
*Cone production*				
Factory B	7.7–9.8	8.6 (± 0.7)	8.6 (± 4.8)	0 (0%)
Factory A	6.8–16	10.7 (± 2.8)	10.3 (± 1.3)	10 (50%)
Factory B	7.3–55.8	11.8 (± 10.7)	10.1 (± 1.8)	5 (25%)
Both Factory A and B	6.8–55.8	11.3 (± 7.7)	10.2 (± 1.4)	15 (37.5%)

Number of participant (*N*); milligram per meter cube (mg/m^3^), *GM* Geometric mean, *SD* Standard deviation, *GSD* Geometric standard deviation, *OEL* Occupational exposure limit

### Observational findings of paper factories

Observational findings of the study revealed that dust had accumulated on the walls, ceilings, floors, and machines of the different working environments. Particularly high dust levels had accumulated in places where the paper-cutting task takes place, such as the bell machine compactor and cone production. Both factories had a dust absorber in the machine (local exhaust system) that pulled excess dust and paper waste away from the process a so-called trim waste system. The employees were wearing medical (surgical) masks; however, these masks were designed to prevent the transmission of Coronavirus, and not for protecting the workers from paper dust exposure. In one paper factory, the machine was very old established more than 60 years. This might increase dust concentration in that work-place. There was no natural or mechanical ventilation. The design and age of the machinery involved, and the absence of natural or mechanical ventilation increased the dust exposure level in these work-places (Additional file 1).

### Prevalence of chronic respiratory symptoms in workers

The prevalence of chronic respiratory symptoms in workers in the paper factories and the controls varied from 17 to 33% and 4–8% respectively. The prevalence of at least one chronic respiratory symptom at a work-place was 51.9% and 9.3% respectively for paper factories and controls. The prevalence ratio of any chronic respiratory symptoms of paper factories was significantly higher than controls after adjusting for sex, age, educational status, monthly income, work experience, working hours per day, working day per week, exposure in previous employments, past history of respiratory illnesses, biofuel energy use, use of PPE, OHS training and OHS supervision (Table 4).

**Table 4:** Prevalence of chronic respiratory symptoms among paper and water bottling factories workers (*N* = 411)

Chronic respiratory symptoms	Paper factories *N* = 206	Control *N* = 205	PR_crude_ (95% CI)^a^	PR_adj_ (95% CI)^a^
Cough *n* (%)	4 1(19.9)	13 (6.3)	3.1 (1.7–5.9) ^**^	4.1 (1.7 – 9.8) ^*^
Phlegm *n* (%)	37 (18)	11 (5.4)	3.4 (1.8–6.4) ^**^	3.1 (1.1 – 8.3) ^*^
Wheezing *n* (%)	39 (18.9)	9 (4.4)	4.3 (2.2–8.7) ^**^	2.9 (1.2—6.8) ^*^
Breathlessness *n* (%)	50 (24.3)	12 (5.9)	4.2 (2.3–7.6) ^**^	3.0 (1.2 – 7.6) ^*^
Nose irritating *n* (%)	67 (32.5)	17 (8.9)	3.9 (2.4–6.4) ^**^	3.2 (1.6 – 6.3) ^**^
Sneezing *n* (%)	64 (31.1)	15 (7.3)	4.3 (2.5–7.2) ^**^	5.2 (2.8 – 9.5) ^**^
Chest pain *n* (%)	35 (17)	10 (4.9)	3.5 (1.8–6.9) ^**^	2.7 (1.0– 7.0) ^*^
At least one chronic respiratory symptom *n* (%)	107 (51.9)	19 (9.3)	5.6 (3.6–8.8) ^**^	6.0 (3.5–10.3) ^**^

*PR* Prevalence ratio, a = 95% confidence interval, * significant level at *p* < 0.05;** = *p* < 0.001; controls were employed in the water bottling factories

### Factors associated with chronic respiratory symptoms among paper factory workers

Among the different characteristics of participants factors considered, educational status was found to be the greatest risk factor to chronic respiratory symptoms. In this study respondents who had not completed secondary school had 4 times (AOR = 3.8, 95% CI 1.5–10.0) higher odds of having chronic respiratory symptoms than those who had completed secondary school (Table 5).

**Table 5:** Factors associated with chronic respiratory symptoms among paper factory workers

Variables	Respiratory symptoms		OR_crude_ (95% CI)	OR_adj_ (95% CI)
	Yes (%)	No (%)		
*Age*				
18–25	25 (23.4)	41 (41.4)	1.0	1.0
26–34	35 (32.7)	32 (32.3)	1.8 (0.9–3.6)	1.4 (0.6–3.3)
≥ 35	47 (43.9)	26 (26.3)	3 (1.5–5.9)*	1.9 (0.8–4.5)
*Educational status*				
1 – 8	25 (23.4)	10 (10.1)	3.5 (1.5–8.0)*	3.8 (1.5–10.0)**
9–12	43 (40.2)	35 (35.4)	1.7 (0.9–3.1)	1.4 (0.8–3.2)
More than 12 years of education	39 (36.4)	54 (54.5)	1.0	1.0
*Monthly income*				
< 4000	85 (79.4)	67 (67.7)	1.9 (1.0–3.5)	2.5(1.1–5.5)**
≥ 4000	22 (20.6)	32 (32.3)	1.0	1.0
*Work section*				
Recycle paper cutting	18 (14.3)	22 (7.7)	8.01 (3. 7–17.5)*	9.5 (1.8–50.8)**
Belling(compactor) and loader	8 (6.3)	3 (1.1)	26.1 (6.4 –106.8)*	46.6 (6.5–353)**
Pulp and recycle paper milling	3 (1.1)	6 (2.1)	4.9 (1.1– 21.2)*	6.4 (0.6–66.2)
Roll paper production	16 (12.7)	7 (2.5)	22.4 (8.2– 61.2)*	43.5 (7.3–257)**
Carton and paper production	49 (38.9)	47 (16.5)	10.2 (5.5- 19)*	16.0 (4.2–61.1)**
Cone production	13 (10.3)	14 (4.9)	9.1 (3.7– 22.1)*	13 (2.6–66.2)**
Water production	19 (15.1)	186 (65.3)	1.0	1.0
*Work experience (in year)*				
< 5	45 (42.1)	57 (57.6)	1.00	1.00
5–9	25 (23.4)	20 (20.2)	1.6 (0. 8– 3.2)	2.7 (1.2–6.4)**
≥ 10	37 (34.6)	22 (22.2)	2.1 (1.1– 4.1)*	3.1 (1.0–9.4)**
*Exposure in previous employments*				
Yes	18 (16.8)	10 (10.1)	1.8 (0.8– 4.1)	1.8 (0.7–4.6)
No	89 (83.2)	89 (89.9)	1.0	1.0
*Past history of respiratory illnesses*				
Yes	31 (29)	6 (6.1)	6.3 (2.5– 16) *	9.3 (3.7–23.8)**
No	76 (71)	93 (93.9)	1.0	1.0

1.00 = reference value, *P < 0.5 for OR_crude_, **P < 0.05 for OR_adj_ OR_crude_ Crude odds ratio, OR_adj_ Adjusted odds ratio

Comparing the different work sections respondents involved in the belling (compactor) and loader section had 47 times (AOR = 46.6, 95% CI 6.2–353) high risk and those in the roll paper production, 44 times (AOR = 43.5, 95% CI 7.3–257), higher risk of having chronic respiratory symptom compared with the control group. The number of years of work in the factory was also significantly associated to the chronic respiratory symptoms. The odds of having chronic respiratory symptom after 5–9 years was 2.7 times higher (AOR = 2.7, 95% CI 1.2–6.4) and for those who had worked more than 10 years, it was 3 times (AOR = 3.1, 95% CI 1.0–9.4) higher compared to workers who had work experience of less than 5 years. The remaining other variables considered were not significantly associated with an increased risk of having chronic respiratory symptoms (Table 5).

## Discussion

This study found that the arithmetic mean (AM (± SD)) and geometric mean (GM (± GSD)) of total dust concentration were 11.3 (± 7.7) and 10.2 (± 1.4) mg/m^3^ respectively, which was above the acceptable recommended limit value of COSHH [31]. The prevalence ratio of all chronic respiratory symptoms among paper factories workers was significantly higher than controls after adjusting for sex, age, religion, educational status, monthly income, work experience, working hours per day, working day per weak, exposure in previous employments, past history of respiratory illnesses, biofuel energy use, use of PPE, and OHS training and supervision. Within the group of paper factory workers a number of factors were significantly associated to at least one chronic respiratory symptom. These included only attaining an educational status below secondary school; working in nearly all work sections of the paper factories; having work experience from 5 to 9 years as well as greater than or equal to 10 years; working more than 8 h/day; and having a past history of occupation and respiratory illnesses.

In the present study, the personal total dust exposure of paper factory workers GM (± GSD)) 10.2 (± 1.4) was consistent with that of two previous studies conducted in Germany, which reported levels of GM (± GSD) 10.3 (± 15.5) and 12.4 mg/m^3^ [8, 9]. The AM of personal dust exposure level in this study was also similar to two studies conducted in Sweden of 9.9 mg/m^3^ and 10.8 mg/m^3^ as well as to a study from Turkey with 9.7 mg/m^3^ [7, 10, 12]. The qualitative findings of our study also support and strengthen this result. Dust levels in the work-place were seen in the accumulations of high amounts dust on the walls, ceiling, floor and machines of the different working environments. In addition, the absence of natural and mechanical ventilation and the presence of very old machines increases dust concentrations [15].

Our study found significantly higher prevalence levels for all chronic respiratory symptoms among the paper factory workers compared to controls. This was similar to a study done in Croatia, where the prevalence of chronic respiratory symptoms such as coughing (36.6% vs. 18.4%) and phlegm (34.7% vs. 16.1%) among workers in paper recycling factories and controls respectively [33]. A similar study conducted in Turkey indicated a prevalence of coughing (31.2% vs. 9%) and wheezing (43.1% vs. 10.3%) among workers in paper factories and controls respectively [10]. As stated previously, these differences might be due to both higher dust exposure levels and low levels of proper PPE use.

The prevalence of chronic respiratory symptoms in this study among paper factory participants (51.9%) was lower than those found in a previous study done in Sweden, (61.5%) [7]. The reason for the difference might be due to the age of the workers in both studies and the sample size difference in the previous study. The Swedish sample size was small and older people were involved. In addition, the difference might be due to the difference in smoking status. In our study, the number of smokers was few.

This study found that demographic characteristics such as educational status and monthly income were significantly associated to the chronic respiratory symptoms among paper factory workers. This study was consistent with a study conducted in Finland, among low socio-economic status, which found that workers with less than secondary school education (AOR = 1.8, 95% CI 1.2–2.6) and having a low income (AOR = 1.4, 95% CI 1.0–1.9) developed more chronic respiratory symptoms compared with those with higher education levels and high-income respectively [34].

Our study found that there was a statistically significant association between chronic respiratory symptoms and duration of employment. Workers with work experience greater than five years had higher chronic respiratory symptoms. This was similar to a study done in Germany, in which the duration of employment for more than 15 had 3 times more symptoms of coughing (AOR = 3.3, 95% CL; 1.4–8.8) compared to less than 15 years duration of employment [8]. This might be directly related to the duration of dust exposure. In other words, longer employment duration means more exposure to dust at the work-place.

### Strength and limitation of the study

This is the first study to assess chronic respiratory symptoms and determine dust exposure levels among paper factory workers in Ethiopia. This study also used control groups to ensure the internal validity of the study.

Generally, the total dust sampling Millipore Plastic Closed-Face Filter Cassette (CFC) significantly underestimated the number of coarse particles in the inhalable paper dust level. There can be lost due to the adherence of dust to the interior part of the cassette walls, which may result in lower values thus underestimating the actual dust concentration.

As this study used interviews to assess chronic respiratory symptoms there might be respondents recall bias and this might have influenced the results. In addition, the cross-sectional study design is not appropriate to show a cause and effect relationship between risk factors and outcomes. It is also important to note that there may be other variables present, that we have not identified. We would therefore recommend a longitudinal study that assesses the effect of PPE, ventilation, work-place cleaning, and new machinery on the development of respiratory symptoms. Moreover, the outcomes could also be underestimated due to the healthy worker’s effect.

## Conclusion

Findings from this study indicate that the AM (± SD) and GM (± GSD) of the concentration of dust workers in the paper factories were 11.3 (± 7.7) and 10.2 (± 1.4) mg/m^3^ respectively. Fifteen (37.5%) dust measurements exceeded OEL value of 10 mg/m^3^ given by COSHH. The prevalence of chronic respiratory symptoms among the paper factory workers was higher than among controls. The main determining factors for chronic respiratory symptoms among the workers were the specific work section such as production sections, the number of years of working, working hours per day and low educational status. We conclude that there should be an effort to reduce dust exposure level in paper factories in Ethiopia to reduce the occupational hazard of workers having chronic respiratory symptoms.

## Supplementary Information

**Additional file 1.** Supplementary material regarding on observational checklist, chronic respiratory symptoms and past history of respiratory illnesses.

## Data Availability

The datasets generated and/or analyzed during the current study are not publicly available due to study participant privacy/consent agreements but are available from the corresponding author on reasonable request.

## Abbreviations

AM: Arithmetic mean
AOR: Adjusted odds ratio
ATS: American thoracic society
COR: Crude odds ratio
COSHH: Control of substances hazardous to health
CI: Confidence interval
GM: Geometric mean
GSD: Geometric standard deviation
OR: Odds ratio
OEL: Occupational exposure limit
OHS: Occupational health and safety
PPE: Personal protective equipment
