# Supplementary material for: Assessment of paper dust exposure and chronic respiratory symptoms among paper factory workers in, Ethiopia; a comparative cross-sectional study
Source: BMC Pulm Med. 2023 Feb 1;23:48. doi: 10.1186/s12890-023-02338-2 (PMC9890692; doi:10.1186/s12890-023-02338-2)
Supplement: Supplementary file 1 — Additional file 1. Supplementary material regarding on observational checklist, chronic respiratory symptoms and past history of respiratory illnesses. [file 12890_2023_2338_MOESM1_ESM.docx]

1. **Supplementary material regarding on observational checklist**

| S/no | Question | Response | | Comments |
| --- | --- | --- | --- | --- |
|  |  | Yes | No |  |
| PPE Usage | | | | |
| 1 | Is required equipment provided and used? |  | |  |
| 2 | Is there a written procedure for the selection, use and maintenance of PPE? |  | |  |
| 3 | Is personal protection utilized only when it is not reasonably practicable to eliminate or control the hazardous substance or process? |  | |  |
| 4 | Are the areas requiring PPE usage properly identified by warning signs? |  | |  |
| Work-place ventilation | | | | |
| 1 | Is the work place well ventilated /free fresh air movements? |  | |  |
| 2 | Is the ventilation system appropriate for the work performed? |  | |  |
| 3 | Is exhaust ventilation required before confined space entry? |  | |  |
| 4 | Is there any Dust absorber |  | |  |
| 5 | Do you ever had occupational health and safety training? |  | | Document observation |

1. **Supplementary material regarding on chronic respiratory symptoms**

| S/N | Question | Response |
| --- | --- | --- |
| 1 | Do you usually have a cough? | 1. Yes 2. No |
| 2 | Do you usually cough with sputum first thing in morning? | 1. Yes 2. No |
| 3 | Do you usually bring up phlegm from your chest as much as twice a day, 4 or more days out of the week? | 1. Yes 2. No |
| 4 | Do you have wheeze/whistling/ sound in your chest? | 1. Yes 2. No |
| 5 | Are you troubled by nose irritation? | 1. Yes 2. No |
| 6 | Are you troubled by sneezing once you start paper factories? | 1. Yes 2. No |
| 7 | Are you troubled with shortness of breath when hurrying or walking uphill | 1. Yes 2. No |

1. **Supplementary material regarding on past history of respiratory illnesses**

| S/N | Question | Response |
| --- | --- | --- |
| 1 | Did you have previous chronic lung conditions told by a doctor? | 1. Yes 2. No |
| 2 | If you say “Yes” the above question what type of health problem did you experienced earlier? (more than one answer possible) | 1. Chronic bronchitis 2. Emphysema 3. Asthma 4. Lung cancer 5. Tuberculosis (TB) 6. Other (specify) |
